# Insights into the Genomic Regions and Candidate Genes of Senescence-Related Traits in Upland Cotton via GWAS

**DOI:** 10.3390/ijms23158584

**Published:** 2022-08-02

**Authors:** Qibao Liu, Zhen Feng, Chenjue Huang, Jia Wen, Libei Li, Shuxun Yu

**Affiliations:** College of Advanced Agriculture Sciences, Zhejiang A&F University, Hangzhou 311300, China; liuqibao566@163.com (Q.L.); fengzhen@zafu.edu.cn (Z.F.); huang_chenjue@163.com (C.H.); 17773326468@163.com (J.W.)

**Keywords:** GWAS, upland cotton, senescence, genomic region, candidate gene, *GhMKK9*

## Abstract

Senescence is the last stage of plant development and is controlled by both internal and external factors. Premature senescence significantly affects the yield and quality of cotton. However, the genetic architecture underlying cotton senescence remains unclear. In this study, genome-wide association studies (GWAS) were performed based on 3,015,002 high-quality SNP markers from the resequencing data of 355 upland cotton accessions to detect genomic regions for cotton senescence. A total of 977 candidate genes within 55 senescence-related genomic regions (SGRs), SGR1–SGR55, were predicted. Gene ontology (GO) analysis of candidate genes revealed that a set of biological processes was enriched, such as salt stress, ethylene processes, and leaf senescence. Furthermore, in the leaf senescence GO term, one candidate gene was focused on: *Gohir.A12G270900* (*GhMKK9*), located in SGR36, which encodes a protein of the MAP kinase kinase family. Quantitative real-time PCR (qRT-PCR) analysis showed that *GhMKK9* was up-regulated in old cotton leaves. Overexpression of *GhMKK9* in *Arabidopsis* accelerated natural leaf senescence. Virus-induced gene silencing (VIGS) of *GhMKK9* in cotton increased drought tolerance. These results suggest that *GhMKK9* is a positive regulator and might be involved in drought-induced senescence in cotton. The results provide new insights into the genetic basis of cotton senescence and will be useful for improving cotton breeding in the future.

## 1. Introduction

Cotton (*Gossypium* spp.) is an important industrial crop worldwide that offers renewable natural fibers, oil, and animal feed [1]. The genomes of the genus *Gossypium* are extraordinarily diverse, including approximately 45 diploid species (2n = 2x = 26) and seven tetraploid (2n = 4x = 52) species [2,3]. *Gossypium hirsutum* L. (also known as upland cotton), one of the seven tetraploid cotton species, is the most widely cultivated species worldwide because of its adaptability, high yield, and moderate fiber quality [4,5]. Although upland cotton makes a significant contribution to revenue in several countries [4], cotton yield is reduced due to senescence when it is induced prematurely under adverse environmental stresses [6].

Senescence is the last stage of plant development and is accompanied by a transition from nutrient assimilation to nutrient remobilization [7,8]. During plant senescence, many major macromolecules are degraded, including proteins, lipids, and nucleic acids, but the most visible symptom is leaf yellowing owing to the catabolism of chlorophyll [9,10]. The onset and progression of senescence are regulated by both internal and external factors. Internal factors include various phytohormones [7,11] that play diverse roles in leaf development. For example, ethylene, abscisic acid (ABA), and salicylic acids (SA) are acknowledged as senescence-promoting hormones [12,13,14,15,16]. Additionally, multiple external environmental factors, including abiotic and biotic stresses, can trigger changes of hormones, which form a complex regulatory network of senescence [8]. Interestingly, the mitogen-activated protein kinase (MAPK) cascades play an important role in conveying endogenous and exogenous signals [17].

Senescence is a complex, quantitative trait, and many studies have reported the genetic basis of leaf senescence in plants. Under various stress conditions, several quantitative trait loci (QTL) associated with senescence were discovered using linkage mapping in crop plants, such as rice [18,19], wheat [20,21,22,23], barley [24], maize [25], sorghum [26,27,28,29], and potato [30]. Although these studies are helpful for understanding the genetic architecture of senescence, it is difficult to identify the underlying genes owing to a lack of resolution. In the past decade, genome-wide association studies (GWAS) have become a powerful method for detecting quantitative trait loci and candidate genes at the genome-wide level [31,32,33,34]. In a recent study, 25 candidate genes for chlorophyll content (CC) and stay-green (SG) traits were identified using a diverse population of 368 rice accessions via GWAS [35]. *OsSG1* is considered a pleiotropic gene regulating CC, SG, and chlorophyll accumulation [35]. In another GWAS study, 64 candidate genes associated with maize senescence were identified using the maize diversity panel, of which 14 genes were involved in senescence-related processes, such as proteolysis and sink activity, and eight candidate genes were supported by a regulatory network [36]. Furthermore, our previous study revealed 50 genomic regions associated with cotton senescence via a multi-locus GWAS based on 185 upland cotton accessions and SLAF-seq data [37]. The candidate gene, *GhCDF1*, was identified as a negative regulator of cotton senescence. However, further studies are needed to understand the mechanisms underlying cotton senescence.

Here, a genome-wide association study was conducted to dissect the genetic basis of senescence in cotton. The association panel consisted of 355 upland cotton accessions planted in multiple environments, and chlorophyll content indices were measured as indicators of senescence. Using resequencing data, 55 senescence-related genomic regions (SGRs) were discovered based on GWAS, and 977 potential candidate genes associated with cotton senescence were identified. The function of candidate gene *GhMKK9* was then analyzed, and it was found that *GhMKK9* silencing improves the drought resistance of cotton, whereas *GhMKK9* overexpression accelerates senescence in *Arabidopsis*. These results provide a foundation for the breeding and the genetic improvement of cotton.

## 2. Results

### 2.1. Analysis of Phenotypic Variations

To evaluate the variability of senescence in the GWAS panel, the relative chlorophyll levels of 355 upland cottons were investigated with the SPAD-502 m during two periods, the flowering and boll-setting period (FBP) and the boll-opening period (BOP), in multiple environments, including Anyang (AY) and Huanggang (HG) in 2016 and 2017, designated as SPAD_FBP_AY16, SPAD_FBP_AY17, SPAD_FBP_HG16, SPAD_FBP_HG17, SPAD_BOP_AY16, SPAD_BOP_AY17, SPAD_BOP_HG16, and SPAD_BOP_HG17. To assess the rate of leaf senescence, the diurnal variation of SPAD was calculated, including D_SPAD_AY1, D_SPAD_AY17, D_SPAD_HG16, and D_SPAD_HG17. Additionally, the absolute chlorophyll concentrations and diurnal variation were determined at AY in 2017 (see the Methods section).

**Figure 1:**
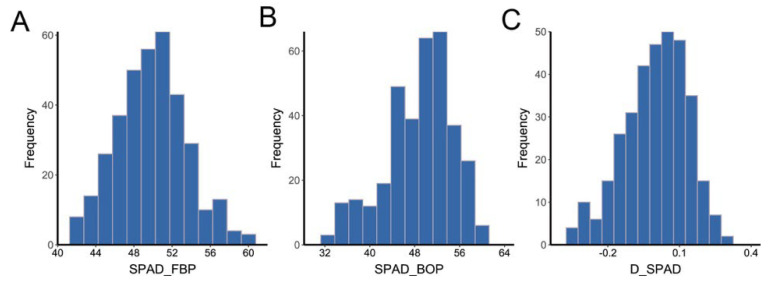
Frequency distributions of the mean values of SPAD. (**A**) The mean value of SPAD in the FBP period. (**B**) The mean value of SPAD in the BOP period. (**C**) The mean value of diurnal variation of SPAD.

The investigated traits followed approximately normal distributions (Figure 1 and Figure S1–S3) and exhibited wide variation among different years and locations (Supplementary Table S1). In the FBP period, the average SPAD values in AY and HG in 2016 were 49.12 and 46.27, respectively, compared to 55.10 and 48.87 in 2017. In the BOP period, the average SPAD in AY in 2016 was higher than that in 2017, at 52.01 and 48.77, respectively, whereas the average SPAD in HG in 2016 was 42.52, lower than that in 2017 (50.19). The standard deviation of SPAD values in the FBP period was distributed from 2.25 to 3.66, compared with the range of 3.54–12.84 in the BOP period. In addition, the average variations of the index D_SPAD ranged from −0.19 to 0.19. Furthermore, the ANOVA result indicated that genotype, environment, and the genotype-by-environment interaction had significant effects on SPAD (*p* < 0.01), while heritability of SPAD in the FBP period was higher than that in the BOP period (0.65 and 0.41, respectively) (Supplementary Table S2). These results indicate that cotton senescence is significantly influenced by environmental factors, particularly in the BOP period.

Pearson’s product–moment correlation coefficients and test statistics were used to evaluate traits. Although there were significant positive correlations (*p* < 0.001) among chlorophyll contents, the diurnal variations of chlorophyll content were more related to the BOP period (|r| = 0.00–0.35) than the FBP period (|r| = 0.01–0.93) (Supplementary Figure S4).

### 2.2. GWAS for Cotton Senescence and Identified Genomic Regions

A total of 3,015,002 high-quality single-nucleotide polymorphisms (SNPs) were identified after a strict filtering pipeline. GWAS was then performed for both single traits across different environments and the best linear unbiased prediction (BLUP) values across all environments using a linear mixed model by EMMAX [38] (Supplementary Figure S5–S7). Given the significant thresholds (*p* < 10^−6^ or *p* < 10^−5^ in at least two environments), 380 significant signals were identified (Supplementary Table S3).

Because the majority of GWAS signals are usually located in noncoding or intergenic regions, functional variations are rarely identified by association tests from SNPs [39]. Therefore, significant signals were integrated, and 55 senescence-related genomic regions (SGRs) obtained, namely, SGR1–SGR55. (Table 1). The total span of SGRs was approximately 18.09 megabases (Mb), of which 27 were over 1 kb in length. In the A subgenome, 37 SGRs were distributed across all 13 chromosomes (A01–A13) with a total length of 9.49 Mb, while 18 SGRs were distributed across only nine chromosomes of the D subgenome, with a total length of 8.60 Mb. Interestingly, there was an extremely long genomic region on the D12 chromosome, SGR52, which spanned 4.33 Mb and accounted for half of the total length of SGRs in the D subgenome. In addition, forty-three SGRs (78.18%) were detected at least twice, indicating that the results were stable and reliable.

### 2.3. Prediction of Candidate Genes

**Figure 2:**
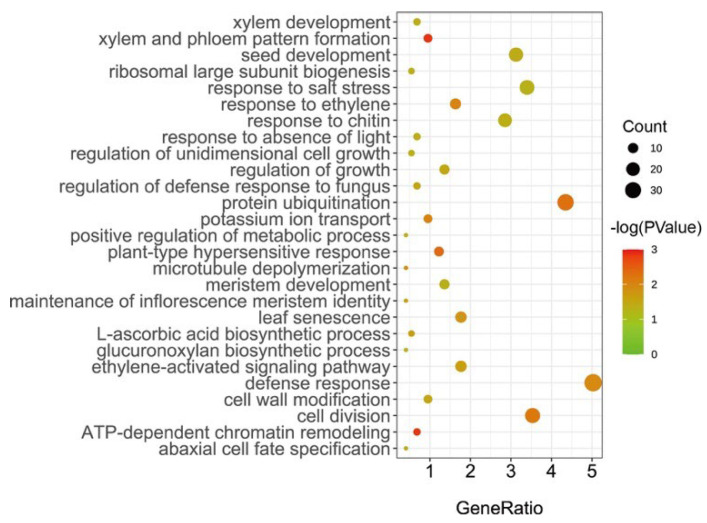
GO enrichment analysis of candidate genes associated with cotton senescence.

In this study, all the genes located in the 55 SGRs were identified as candidate senescence-related genes. Subsequently, 977 candidate genes were identified (Supplementary Table S4). Of these, 853 candidate genes were annotated as orthologs in *Arabidopsis*. Notably, 156 genes were recorded in the leaf senescence database LSD 3.0, such as *EIN3* (*Gohir.A03G034800*/*Gohir.A03G034800*), *WRKY6* (*Gohir.D07G088100*), and *PPH* (*Gohir.D12G102900*) (Supplementary Table S5). This result suggests that our approach to dissecting the genetic basis of cotton senescence was effective. Furthermore, enrichment analysis of gene ontology (GO) biological processes (BPs) showed that the significant enrichments (*p* < 0.05) of these genes were associated with plant senescence-related processes, such as response to salt stress, ethylene processes, and leaf senescence (Figure 2). For example, *Gohir.D12G208700* (*GhRCD1*) is a homolog of *AT1G32230* in *Arabidopsis*, encoding a protein belonging to the (ADP-ribosyl) transferase domain-containing subfamily of the WWE protein–protein interaction domain protein family, and RCD1 was reported to be involved in superoxide-induced cell death [40,41]. *Gohir.A12G270200* (*GhJAZ3*) encodes jasmonate zim-domain protein 3, which negatively regulates *AtMYC2*, a key transcriptional activator of JA responses [42]. Most strikingly, we focused on the candidate gene *Gohir.A12G270900* (*GhMKK9*), which is a homolog of *AT1G73500* (*AtMKK9*), a member of the MAP kinase kinase family that was reported to play a positive role in leaf senescence of *Arabidopsis* [43].

**Figure 3:**
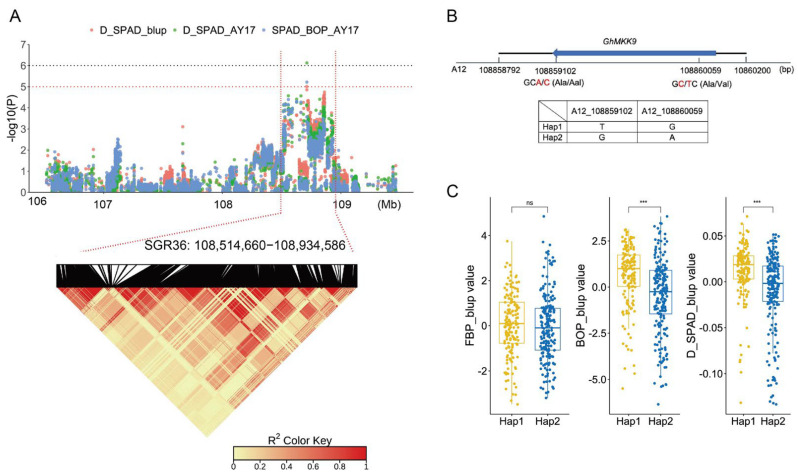
GWAS identification of candidate gene in the SGR36. (**A**) Manhattan plot (upper) and LD heat map (lower) of SGR36. (**B**) Gene structure and haplotypes of the candidate gene *GhMKK9*. (**C**) Phenotypes of different haplotypes. There are 158 accessions for Hap1 and 197 accessions for Hap2. Asterisks indicate significance levels (*** *p* < 0.001); ns, not significant.

*GhMKK9* is located in SGR36, which spans approximately 420 kb and is associated with three phenotypic values, D_SPAD_AY17, SPAD_BOP_AY17, and D_SPAD_blup (Figure 3A). In the genomic region, we discovered a non-synonymous SNP (A12_108859102) within the CDS region of *GhMKK9*, which causes a change in the base from C to T, as well as a change in amino acid from alanine (GCC) to valine (GTC) (Figure 3B). This SNP and another synonymous SNP (A12_108860059), also located in the CDS region, form two haplotypes, TG (Hap1) and GA (Hap2). In the associated panel, 158 cotton accessions carried Hap1, and 197 accessions carried Hap2. Although the SPAD values (FBP_blup) of Hap1 and Hap2 were not significantly different in the FBP period, the BOP_blup and D_SPAD_blup values of Hap1 were significantly higher than those of Hap2 (Figure 3C), indicating that Hap1 is a favorable haplotype for delaying cotton senescence.

### 2.4. GhMKK9, A Positive Regulator of Cotton Senescence

**Figure 4:**
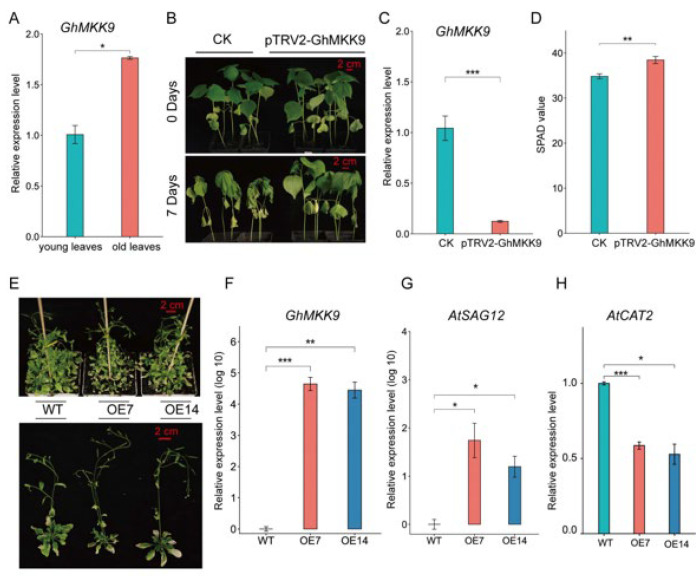
Functional analysis of the candidate gene *GhMKK9*. (**A**) Expression of *GhMKK9* in young and old cotton leaves by qRT-PCR. (**B**) Phenotypes of empty control (CK) and VIGS cotton plants (pTRV2-GhMKK9) under drought stress. After four weeks, the CK and VIGS cotton plants were treated with water shortage for 7 days. (**C**) Expression levels of *GhMKK9* in the CK and VIGS cotton plants. (**D**) SPAD value of the CK and VIGS plants under drought stress. (**E**) Phenotypes of six-week-old WT and transgenic *Arabidopsis* plants (OE7 and OE14). (**F**) Expression levels of *GhMKK9* in the WT and transgenic *Arabidopsis* plants. (**G**,**H**) Expression levels of senescence-marked genes *AtSAG12* and *AtCAT2* in the WT and transgenic *Arabidopsis* plants. Asterisks indicate significance levels (*** *p* < 0.001, ** *p* < 0.01, and * *p* < 0.05).

Quantitative real-time PCR (qRT-PCR) analysis showed that the expression level of *GhMKK9* in old cotton leaves was significantly higher than that in young cotton leaves (Figure 4A). Furthermore, we silenced the expression of *GhMKK9* in cotton using virus-induced gene silencing (VIGS) (Figure 4C). After one week of drought treatment, the CK group showed an obvious leaf wilting phenotype, whereas the VIGS-silenced plants (pTRV2-GhMKK9) only showed a barely visible wilting phenotype (Figure 4B). The SPAD value of cotton leaves in the CK group after drought treatment was also significantly lower than that of the VIGS-silenced plants (Figure 4D). Moreover, to further examine the function of *GhMKK9*, we overexpressed *GhMKK9* under the control of the 35S promoter (35S::GhMKK9) in *Arabidopsis* and obtained two transgenic lines (OE7 and OE14), which were confirmed by qRT-PCR (Figure 4F). After six weeks of culture under normal conditions, the overexpressing *Arabidopsis* lines OE7 and OE14 exhibited more severe senescence phenotypes than wild-type *Arabidopsis*, such as rosette leaf wilting and a higher degree of yellowing (Figure 4E). In addition, we determined the transcript levels of two senescence-marked genes, *AtSAG12* (up-regulated during senescence) [44,45] and *AtCAT2* (down-regulated during senescence) [46,47]. The transcript level of *AtSAG12* in the transgenic plants was significantly higher than that in the WT plants (Figure 4G), whereas the transcript level of *AtCAT2* in the transgenic plants was significantly lower than that in the WT plants (Figure 4H). Taken together, these results suggest that *GhMKK9* is a positive regulator of leaf senescence and may also be involved in drought-stress-induced senescence.

## 3. Discussion

The senescence process of plant leaves is a very complex biological regulation process which first depends on age and is also affected by external environmental signal stimuli [11]. Therefore, internal genetic and external environmental factors together determine the onset and rate of senescence. The senescence process in plants involves the remobilization and reutilization of nutrients from senescing parts as sinks [7,48], which is particularly important for crop plant products. Cotton fiber is one of the most important industrial textile fibers worldwide. Senescence has an important impact on the quality and yield of cotton fiber [6]. Compared with other crops, such as rice, wheat, and corn, cotton has the habit of indeterminate growth, which blurs the lines between growth, maturation, and senescence. Nevertheless, the flowering and boll period (FBP) is considered to be an important developmental stage of cotton because the plant undergoes a transition from vegetative to reproductive growth in which the level of plant endogenous hormones reaches a peak, photosynthesis is enhanced, and the activity of the “sink” is also enhanced. Then, in the boll-opening period (BOP), cotton senescence, such as chlorosis, is visible. Therefore, these two periods were chosen to study the regulation of senescence in cotton. Although senescence has received increasing attention in cotton breeding, research on the genetic basis of cotton senescence remains limited. In this study, chlorophyll content indices were selected as indicators to evaluate the senescence performance of the upland cotton population. Due to the combined action of genetic and environmental factors, the chlorophyll content varied widely across different planting locations and years. The SPAD value in the BOP period had a larger range of variation than that in the FBP period. Moreover, the SPAD value in the FBP period showed higher heritability than that in the BOP period (0.65 and 0.41, respectively), which is similar to the results of the previous study [37]. These results indicate that environmental factors have a more significant impact on later cotton development.

**Figure 5:**
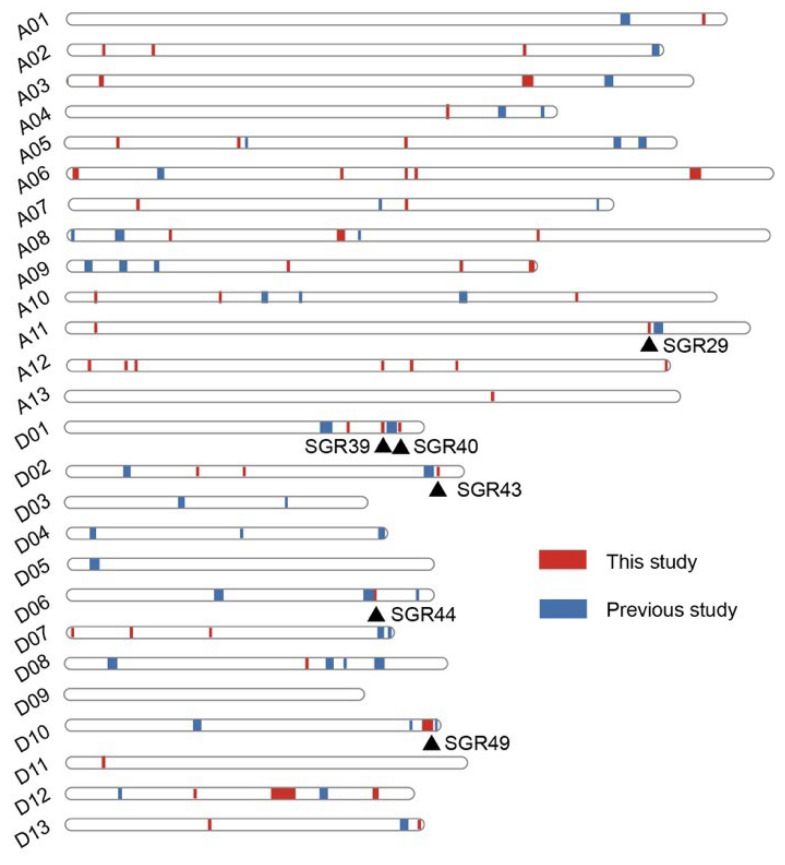
Distribution of senescence-related genomic regions at chromosomes from this and previous studies. Red vertical bars represent genomic regions from this study. Blue vertical bars represent genomic regions from previous study. Black triangles indicate SGRs located within ~1 Mb of the genomic regions reported by previous study.

A GWAS was performed based on 3,015,002 high-quality SNP markers from the resequencing data of 355 accessions to detect the genetic structure of cotton senescence. A total of 380 significant signals were identified. Given that functional variations are usually rare in GWAS [49], significant SNPs were integrated into genomic regions (GWAS loci). In the previous study, 50 genomic regions associated with cotton senescence were revealed based on SLAF-seq data of 185 accessions, which spanned a total of 51.50 Mb [37]. In the present study, 55 senescence-related genomic regions (SGRs) spanning approximately 18.09 Mb were identified. Compared with SLAF-seq-based GWAS, the resequencing data greatly increased the fine-mapping resolution. Six SGRs (SGR29, SGR39, SGR40, SGR43, SGR44, and SGR49) were located within ~1 Mb of the genomic regions reported in the previous study. (Figure 5). Interestingly, these SGRs were located in the D subgenome (except for SGR29) and were associated with the chlorophyll content in the BOP period and/or the diurnal variation of chlorophyll content (excepted for SGR39). These results suggest that the D subgenome plays an important role in the regulation of senescence in cotton. A range of abiotic and biotic stressors, such as drought, salt, and pathogen infection, can accelerate the onset and/or progression of plant senescence [7,8,50], and the D subgenome was reported to make an important contribution to stress tolerance in allotetraploid cotton [51]. This provides a possible explanation for the results.

Of the 55 SGRs, a total of 977 candidate genes were annotated. Among them, 156 genes were also recorded in the leaf senescence database LSD 3.0, and GO analysis revealed a set of biological processes, such as salt stress, ethylene processes, and leaf senescence. This suggests that the theory used in this study was effective. Interestingly, focus was given to a candidate gene, *Gohir.A12G270900*, which is homologous to *AT1G73500* and encodes an MKK9 protein in *Arabidopsis*. *AtMKK9* plays an important role in the regulation of *Arabidopsis* senescence [43]. There are many signaling pathways in plants that involve responses to external stimuli, and one of the most common is the MAPK signaling pathway. In eukaryotes, the MAPs cascade signaling pathway is a highly conserved signaling module [52,53]. Each MAPKs cascade signaling module is composed of three protein kinases that act in sequence: MPK, MKK, and MKKK. In *Arabidopsis*, there are 20 MPK genes, 10 MKK genes, and 69 MKKK genes [54]. In upland cotton, there may be 52 GhMKs, 23 GhMKKs, and 166 GhMKKKs genes [55]. The candidate gene *Gohir.A12G270900* (*GhMKK9*) is a member of the GhMKK family. *GhMKK9* is located in SGR36, which is associated with multiple senescence phenotypes, indicating high repeatability and reliability. Interestingly, a non-synonymous SNP (A12_108859102) and synonymous SNP (A12_108860059) were observed in the exon region of *GhMKK9*. The SNP A12_108859102 changed the amino acid from alanine (GCC) to valine (GTC), which may affect the function of the GhMKK9 protein. In addition, these two SNPs formed two haplotypes, Hap1 and Hap2 (Figure 3C). The BOP_blup and D_SPAD_blup values of the Hap1 are significantly higher than those of the Hap2. These results suggest that Hap1 is a favorable haplotype for delaying senescence and that the *GhMKK9* gene may play an important role in the regulation of cotton senescence.

The function of the *GhMKK9* gene was further verified. By qRT-PCR analysis, it was found that the expression level of *GhMKK9* was significantly higher in old cotton leaves than that in young cotton leaves, and overexpression of *GhMKK9* gene in *Arabidopsis thaliana* promoted the senescence process of *Arabidopsis* leaves, indicating that *GhMKK9* is a positive regulator of plant senescence, which is consistent with the results of a previous study [43,56]. In *Rosa hybrida*, *RhMKK9* silencing significantly delayed petal senescence in flowers [57]. The MKK9–MPK6 module was reported to play an important role in the regulation of the senescence process [43], in which MPK3/MPK6 could be activated by MKK9 to induce ethylene biosynthesis [56,57,58]. Furthermore, the endogenous *GhMKK9* gene in cotton was silenced using VIGS. *GhMKK9* gene-silenced plants were found to have enhanced drought tolerance compared with the control plants (CK), indicating that *GhMKK9* may be involved in drought-stress-induced senescence in cotton. MKK9 is widely involved in the transmission of environmental signals, but its effects on plant stress tolerance remain controversial. For example, Yoo et al. [59] and Shen et al. [60] showed that *AtMKK9* is a positive regulator of salt tolerance in *Arabidopsis*, which is contrary to the results reported by Alzwiy and Morris [61] and Xu et al. [56]. Similarly, although there is no obvious difference between WT and *mkk9* mutant *Arabidopsis* plants under drought stress [61], this study shows that the silencing of *GhMKK9* enhances drought tolerance in cotton. These discrepant results may be attributed to different experimental methods and functionally redundant genes [60].

## 4. Materials and Methods

### 4.1. Plant Materials

The association mapping panels consisted of 355 upland cotton accessions (Supplementary Table S6), and the germplasm resources were obtained from the Institute of Cotton Research of Chinese Academy of Agricultural Sciences (ICR-CAAS). These materials are geographically widespread across China, including in the Yellow River Region (YRR), the Yangtze River Region (YZRR), the Northwest Inland Region (NIR), and the Northern Specific Early-Maturity Region (NSER), and a few were from abroad (e.g., the United States) [62]. In 2016 and 2017, 355 upland cotton accessions were planted in Anyang (AY), Henan (36°08′ N, 114°48′ E), and Huanggang (HG), Hubei (31°14′ N, 114°78′ E), respectively. Three replicates were planted in each environment, except Anyang in 2017, where two replicates were used.

### 4.2. Phenotyping and Data Analysis

The relative chlorophyll level (SPAD) of association panels was measured with the chlorophyll meter SPAD-502 (Konica Minolta, Japan) in four environments in the flowering and boll-setting period (FBP) and boll-opening period (BOP). The third parietal leaf from the top was selected after topping to measure the chlorophyll level, and the average SPAD of at least three individuals for each accession was recorded. The absolute chlorophyll concentration of the materials planted in Anyang in 2017 was also measured. Three discs of 0.6 cm diameter were cut by punch from the third parietal leaf, and these leaf discs were mixed from at least three individuals for each accession. Chlorophyll concentration was estimated using the method described by Arnon [63]. Four chlorophyll concentration indices were obtained: chlorophyll a (Chla), chlorophyll b (Chlb), total chlorophyll (Total_ab), and chlorophyll a/b (Ratio_ab). In addition, the diurnal variation of chlorophyll content was calculated using the following formulae: D (%) = (chlorophyll content of BOP − chlorophyll content of FBP)/(chlorophyll content of BOP × days between FBP and BOP) × 100%, which included D_SPAD, D_chla, D_chlb, D_total_ab, and D_ratio_ab.

The best linear unbiased predictions (BLUPs) and broad-sense heritability (*H*^2^) of SPAD values in the four environments were calculated using the R package sommer [64]. Broad-sense heritability was defined as *H*^2^ = σ_g_^2^/(σ_g_^2^ + σ_gl_^2^/l + σ_gy_^2^/y + σ_e_^2^/rly), where σ_g_^2^ is the genotypic variance; σ_gl_^2^ is the interactions of genotype with location; σ_gy_^2^ is the interactions of genotype with year; σ_e_^2^ is the error variance; and l, y, and r are the number of locations, years, and replications, respectively. Statistical and correlation analyses were performed using the R package Hmisc [65] and visualized using the package corrplot [66].

### 4.3. SNP Genotyping

The resequencing data of 355 upland cotton accessions were reported in a previous study [62]. The quality of paired-end reads from 355 accessions was evaluated using FastQC v.0.11.9 [67] and was controlled using Trimmomatic v.0.39 [68]. All high-quality clean reads were mapped to the *Gossypium hirsutum* v1.1 reference genome [69] with BWA mem v.0.7.17 [70]. The mapping results were sorted and converted to the BAM format using Picard tools (http://broadinstitute.github.io/picard). GATK v.4.1.8 [71] was used to detect variants following the best-practice workflows. High-quality SNPs were filtered with: “QD < 2.0 QUAL < 30.0 FS > 60.0 MQ < 40.0 MQRankSum < −12.5 ReadPosRankSum < −8.0”, missing rate < 50%, and MAF > 0.05.

### 4.4. GWAS and Identification of Genomic Regions

A linear mixed model was used to perform GWAS on 355 upland cotton accessions, implemented in the EMMAX software [38]. Before conducting the GWAS, the SNPs were imputed using Beagle v.5.1 [72]. Both trial values of the single environment and BLUPs were used for the GWAS. Because a high correlation between SNPs always leads to information redundancy, PLINK was used to detect the number of genome-wide, effective SNPs. The parameters for pruning were as follows: within a 500 bp sliding window, r^2^ ≥ 0.2, and a step of 100 bp. After pruning, 925,819 SNPs were obtained, and the genome-wide significance cutoff for GWAS was selected as *p* = 1 × 10^−6^ (1/925819). Significant SNPs were then determined using the following criteria: (1) *p* < 10^−6^ or (2) *p* < 10^−5^ in at least two environmental trail values owing to the stability. To identify senescence-related genomic regions (SGRs), we selected independent, significant SNPs (r^2^ < 0.6). If r^2^ > 0.1, the SNP with *p* < 10^−3^ and independent significant SNP were merged into the same genomic region. In addition, if the distance between two genomic regions was less than 900 kb, they were merged into one genomic region. The R packages CMplot [73], LDheatmap [74], and ggplot2 [75] were used to visualize the GWAS results.

### 4.5. Prediction of Candidate Genes

All genes located in SGRs were selected as putative candidate genes based on the *Gossypium hirsutum* v1.1 reference genome [69]. Homologs of these genes in *Arabidopsis thaliana* were determined using BLAST [76], and GO enrichment was performed on the database for annotation, visualization, and integrated discovery (DAVID) to identify enriched biological themes [77,78].

### 4.6. RNA Extraction and qRT-PCR

To determine the expression level of *GhMKK9*, the cotton accession “CRI 10” was planted in a greenhouse, and two-week-old (young) and eight-week-old (old) leaves were sampled from eight individuals with three biological replicates in each group. Total RNA was extracted using an RNA Purification Kit (Tiangen, Beijing, China), and the RNA was reverse transcribed using the PrimeScript RT Reagent Kit (TAKARA, Dalian, China) following the manufacturer’s instructions. Quantitative real-time PCR (qRT-PCR) was performed on a Roche Applied Science LightCycler 480 using the NovoStart^®^ SYBR qPCR SuperMix Plus (Novoprotein, Shanghai, China). The qRT-PCR was conducted as follows: pre-denaturation at 95 °C for 60 s; 40 cycles of 95 °C for 20 s and 60 °C for 60 s. Three technical replicates were performed for each sample, and the relative expression of genes was calculated using the 2^−ΔΔCt^ method [79]. The primers are listed in Supplementary Table S7.

### 4.7. VIGS

For the VIGS assays, one fragment of *GhMKK9* amplified from the cDNA of “CRI 10” was integrated into the pTRV2 vector (pTRV2-GhMKK9) using the nimble cloning method [80] and then the recombinant vector was introduced into *Agrobacterium tumefaciens* GV3101. *Agrobacterium* strains harboring the pTRV2-GhMKK9 and pTRV2 (negative control) vectors combined with strains harboring the pTRV1 vector were co-transferred into the cotyledons of 2-week-old cotton plants following previously described methods [81]. The injected plants were kept in darkness for 24 h and transferred to a greenhouse at 25 °C with 16 h light/8 h dark cycle. Four weeks after injection, plants injected with pTRV2 and pTRV2-GhMKK9 were subjected to drought treatment, and SPAD values were determined. The primers used for the construction of the VIGS vector and qRT-PCR are listed in Supplementary Table S7.

### 4.8. Genetic Transformation of Arabidopsis Thaliana

The ORF of *GhMKK9* was inserted into the binary expression vector pNC-Cam2304 to generate the 35S::GhMKK9 construct using the nimble cloning method [80]. The 35::GhMKK9 construct was introduced into *Agrobacterium tumefaciens* GV3101 and then transformed into *Arabidopsis* ecotype Columbia using the floral dip method [82]. The positive plants were screened out using 1/2 MS medium containing kanamycin (100 mg/L) and confirmed via qRT-PCR. The T_3_ homozygous generation plants were used for phenotypic observation of senescence. To observe the performance of transgenic plants under normal conditions, seeds of WT and two independent 35S::GhMKK9 lines (OE7 and OE14) were germinated on 1/2 MS agar medium. After two weeks, the seedlings were transplanted into the soil. Phenotypic characteristics were observed, and the rosette leaves at position six from six-week-old plants were sampled for qRT-PCR. Primers used for the construction of 35::GhMKK9 and qRT-PCR are listed in Supplementary Table S7. The primer specificity of GhMKK9 were confirmed (Supplementary Figure S8).

## Figures and Tables

**Table 1:** Summary of senescence-related genomic regions.

SGR	Chr	Start (bp)	End (bp)	Trait
SGR1	A01	115,568,753	115,568,865	Ratio_ab_FBP
SGR2	A02	6,556,304	6,653,102	SPAD_BOP_AY17, D_SPAD_blup, D_SPAD_AY17
SGR3	A02	155,73,340	15,573,343	D_total_ab, D_chla
SGR4	A02	82,886,748	82,925,332	SPAD_BOP_AY16, D_SPAD_AY16
SGR5	A03	5,877,926	6,672,551	D_SPAD_blup, SPAD_BOP_AY17
SGR6	A03	82,626,806	84,562,267	SPAD_BOP_AY16, D_SPAD_AY16
SGR7	A03	113,682,897	113,683,008	SPAD_BOP_AY16, D_SPAD_AY16
SGR8	A04	69,311,017	69,315,686	Ratio_ab_BOP
SGR9	A05	9,695,614	9,695,614	Ratio_ab_FBP
SGR10	A05	31,628,900	31,628,910	SPAD_BOP_AY17, D_SPAD_AY17
SGR11	A05	61,940,854	61,940,864	D_chlb, D_ratio_ab
SGR12	A06	1,128,396	2,179,285	Chlb_BOP, D_chlb, Total_ab_BOP, Ratio_ab_BOP, D_ratio_ab
SGR13	A06	49,902,382	49,941,259	D_ratio_ab, D_chla, D_total_ab, Chla_BOP, Total_ab_BOP
SGR14	A06	61,589,693	61,589,748	Ratio_ab_FBP
SGR15	A06	63,390,768	63,390,795	D_chla, Chla_BOP, Total_ab_BOP
SGR16	A06	113,048,017	115,046,117	D_SPAD_HG17, Ratio_ab_BOP
SGR17	A07	12,602,257	12,602,280	SPAD_BOP_AY17, D_SPAD_AY17
SGR18	A07	61,313,631	61,313,636	Chla_BOP, Total_ab_BOP
SGR19	A08	18,726,393	18,727,072	D_SPAD_AY16, SPAD_BOP_AY16
SGR20	A08	48,942,300	50,338,088	SPAD_BOP_AY17, D_SPAD_AY17
SGR21	A08	85,414,259	85,415,664	Ratio_ab_BOP, D_SPAD_HG17
SGR22	A09	40,190,612	40,190,642	D_SPAD_AY17, D_SPAD_blup
SGR23	A09	71,543,578	71,543,578	Ratio_ab_FBP
SGR24	A09	83,855,283	84,822,913	D_SPAD_AY16, SPAD_BOP_AY16
SGR25	A10	5,521,694	5,526,321	D_SPAD_AY16, SPAD_BOP_AY16
SGR26	A10	27,875,320	28,364,565	BOP_blup, D_SPAD_blup
SGR27	A10	92,752,436	92,766,905	SPAD_FBP_HG16, Ratio_ab_FBP
SGR28	A11	5,387,130	5,588,967	D_SPAD_blup, D_SPAD_AY17, SPAD_BOP_AY17
SGR29	A11	106,469,948	106,477,914	D_SPAD_AY16, SPAD_BOP_AY16
SGR30	A12	4,116,205	4,126,358	Chlb_FBP, Total_ab_FBP
SGR31	A12	10,742,829	10,742,829	FBP_blup
SGR32	A12	12,591,095	12,594,279	D_SPAD_blup, D_SPAD_AY17, SPAD_BOP_AY17
SGR33	A12	57,300,054	57,307,338	D_SPAD_AY16, SPAD_BOP_AY16
SGR34	A12	62,551,488	62,556,405	D_ratio_ab, D_ratio_ab
SGR35	A12	70,740,474	70,740,474	D_SPAD_blup, D_ratio_ab, D_SPAD_AY17
SGR36	A12	108,514,660	108,934,586	SPAD_BOP_AY17, D_SPAD_AY17, D_SPAD_blup
SGR37	A13	77,580,822	77,580,838	BOP_blup, SPAD_BOP_AY16
SGR38	D01	51,414,839	51,415,298	BOP_blup, D_SPAD_blup
SGR39	D01	57,675,353	57,675,364	Ratio_ab_FBP, Chla_FBP
SGR40	D01	60,671,107	60,671,202	D_SPAD_blup, D_SPAD_AY17
SGR41	D02	23,837,463	23,837,463	Ratio_ab_FBP
SGR42	D02	32,314,480	32,314,480	D_SPAD_AY17, SPAD_BOP_AY17
SGR43	D02	67,476,213	67,476,218	D_ratio_ab
SGR44	D06	55,810,689	56,177,650	Ratio_ab_BOP, D_ratio_ab, D_SPAD_HG17
SGR45	D07	510,384	510,546	Ratio_ab_BOP
SGR46	D07	10,915,964	11,432,705	SPAD_FBP_HG17, SPAD_FBP_HG17
SGR47	D07	26,125,297	26,125,366	Ratio_ab_FBP, D_ratio_ab, Chlb_FBP
SGR48	D08	43,939,104	43,939,104	Chlb_FBP
SGR49	D10	65,317,192	67,265,400	SPAD_BOP_AY17, BOP_blup, D_SPAD_AY17, FBP_blup, SPAD_FBP_HG16
SGR50	D11	9,657,009	10,060,413	FBP_blup, SPAD_FBP_HG16
SGR51	D12	23,540,471	23,540,583	D_SPAD_AY16
SGR52	D12	37,359,379	41,685,124	D_total_ab, Chla_BOP, BOP_blup, SPAD_BOP_HG16, SPAD_FBP_HG16
SGR53	D12	55,716,433	56,755,391	Ratio_ab_BOP, D_ratio_ab
SGR54	D13	26,198,781	26,198,804	D_SPAD_AY17, SPAD_BOP_AY17
SGR55	D13	64,827,390	64,827,424	Ratio_ab_FBP

SGR: senescence-related genomic region; Chr: chromosome.

## Data Availability

The datasets presented in this study can be found in online repositories. The names of the repository/repositories and accession number(s) can be found in the Supplementary Materials.

## Supplementary Materials

The following supporting information can be downloaded at: https://www.mdpi.com/article/10.3390/ijms23158584/s1.
