# Supplementary material for: Insights into the Genomic Regions and Candidate Genes of Senescence-Related Traits in Upland Cotton via GWAS
Source: Int J Mol Sci. 2022 Aug 2;23(15):8584. doi: 10.3390/ijms23158584 (PMC9368895; doi:10.3390/ijms23158584)
Supplement: Supplementary file 1 [file ijms-23-08584-s001.zip › Supplementary Figrue S1¿CS8.pdf]

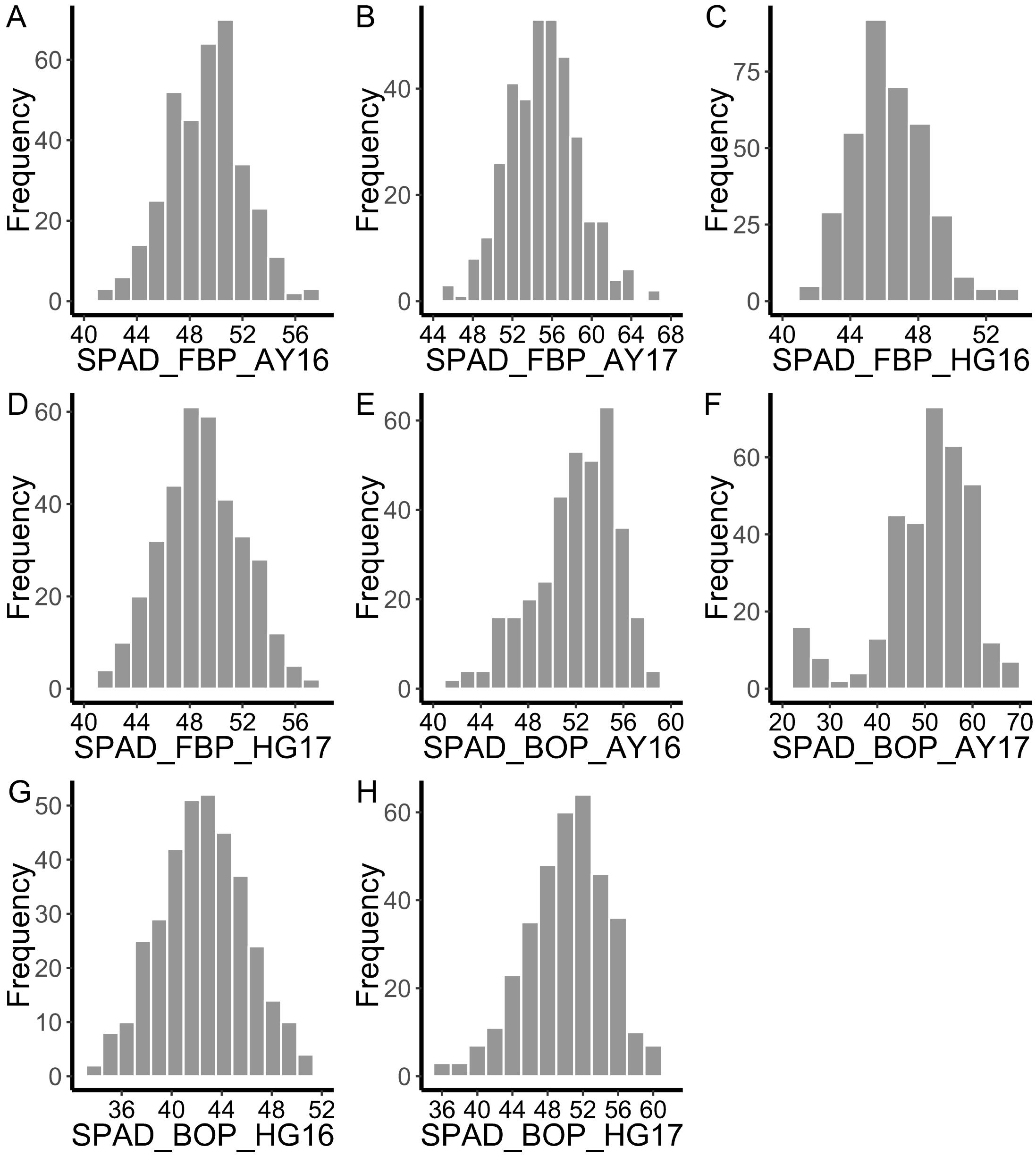

Figure S1. Frequency distributions of SAPD in each single environment. FBP: he flowering and boll-setting period; BOP: the boll opening period; AY: Anyang; HG: Huanggang; 16: 2016; 17: 2017.

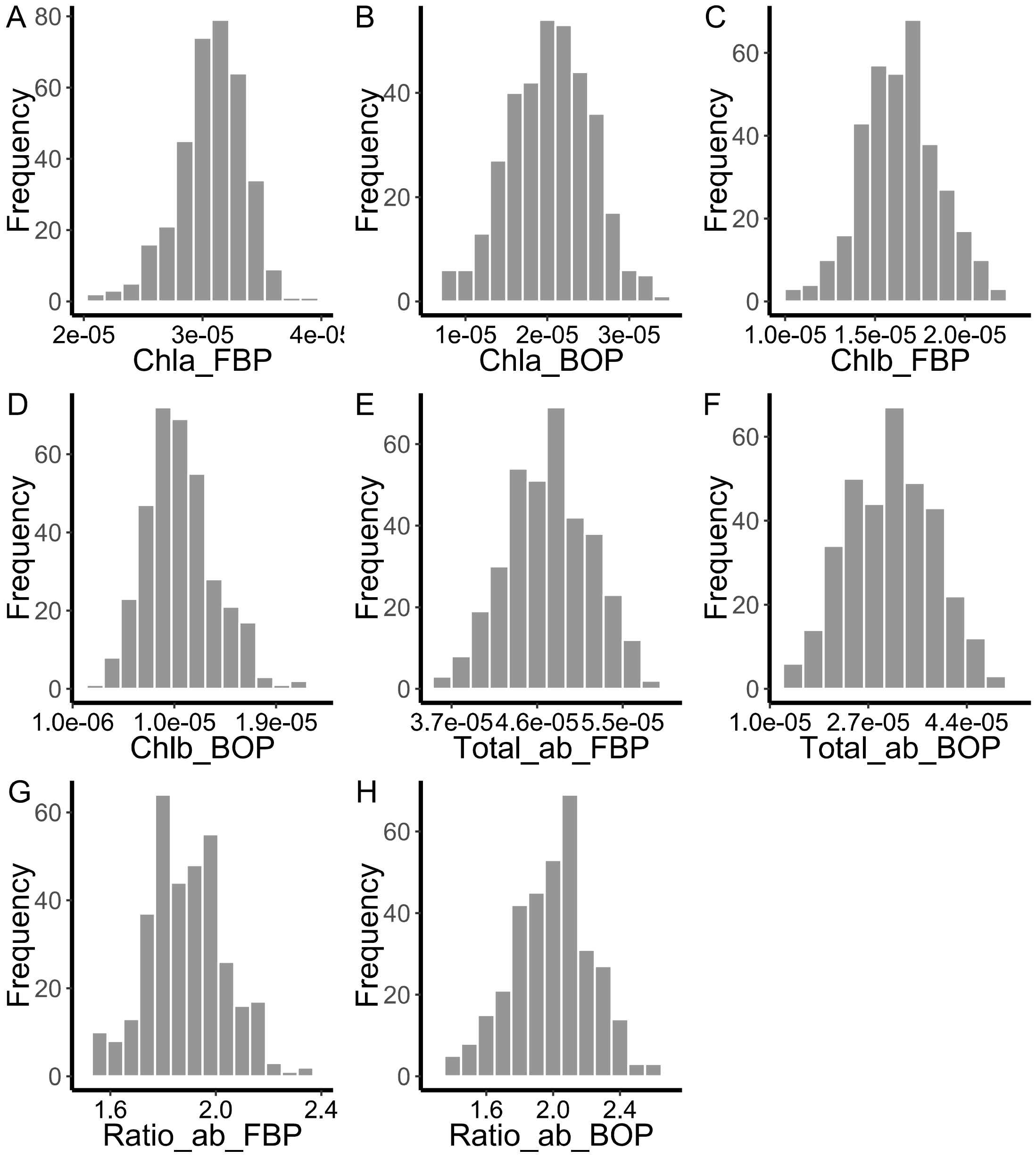

Figure S2. Frequency distributions of absolute chlorophyll concentrations at Anyang in 2017. Chla: Chlorophyll a; Chlb: Chlorophyll b; Total_ab: Total chlorophyll; Ratio_ab: Chlorophyll a/b; FBP: The flowering and boll-setting period; BOP: the boll opening period.

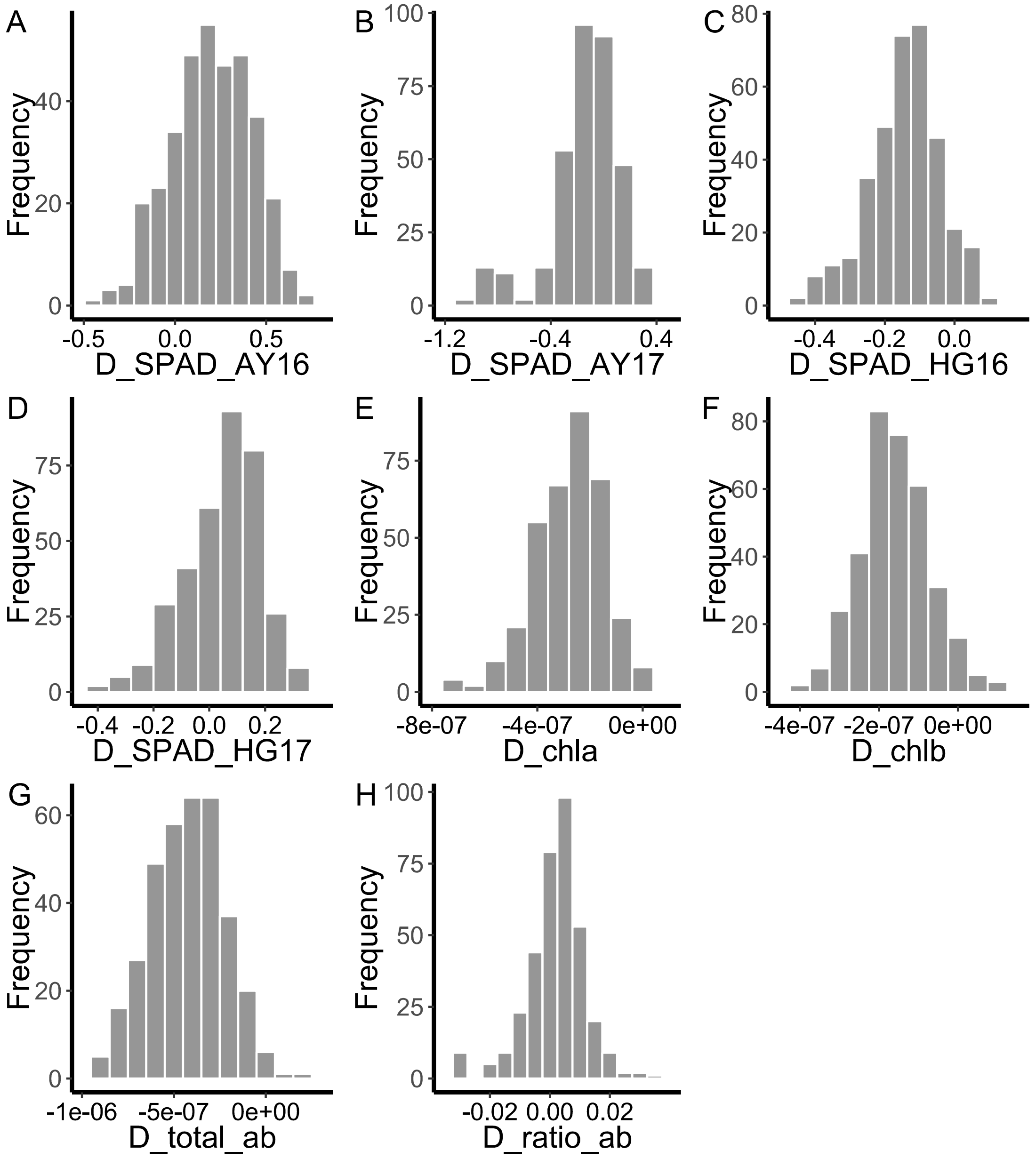

Figure S3. Frequency distributions of the diurnal variation of chlorophyll content. D: the diurnal variation; Chla: Chlorophyll a; Chlb: Chlorophyll b; Total_ab: Total chlorophyll; Ratio_ab: Chlorophyll a/b; FBP: The flowering and boll-setting period; BOP: the boll opening period; AY: Anyang; HG: Huanggang; 16: 2016; 17: 2017.

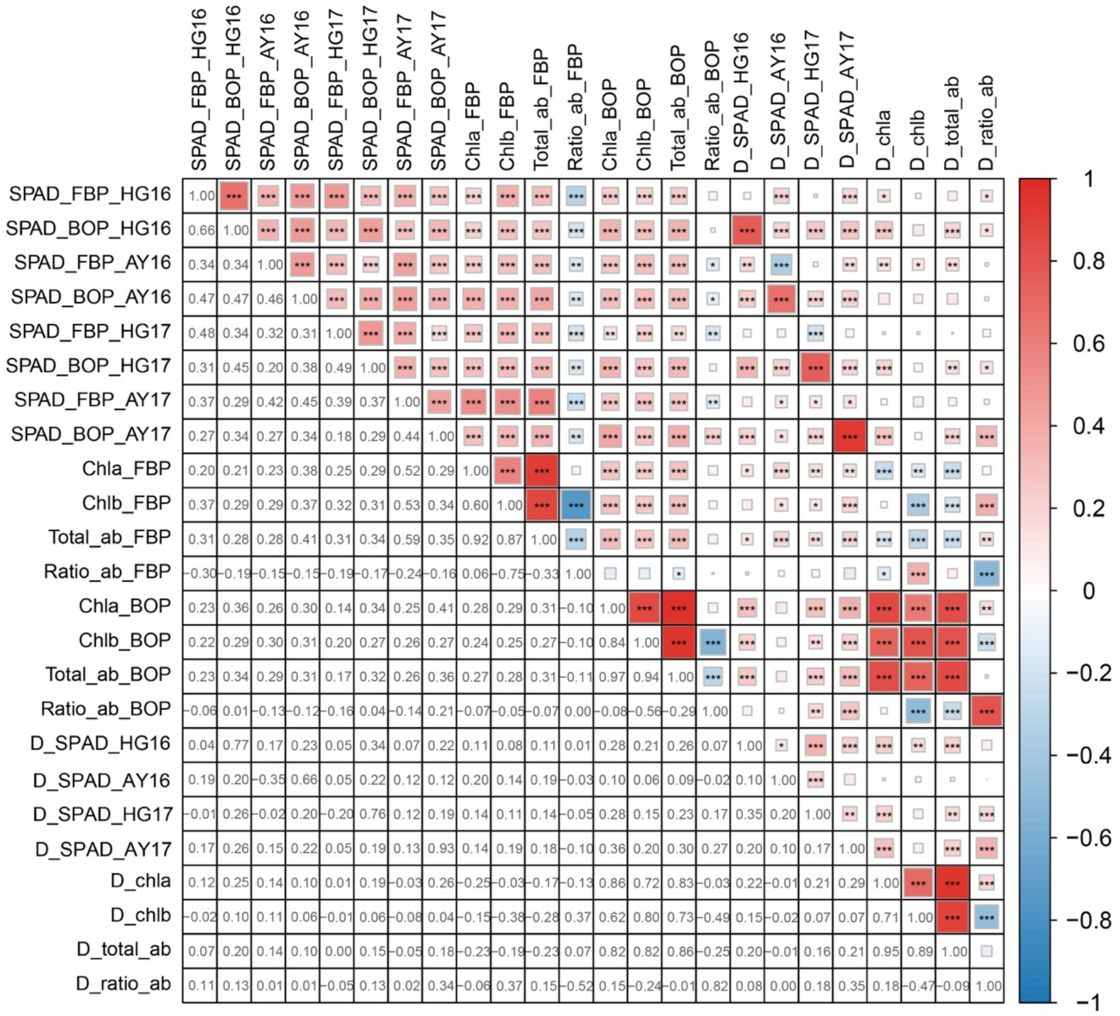

Figure S4. Correlation analyses between indices of chlorophyll content in multi-environments. D: the diurnal variation; Chla: Chlorophyll a; Chlb: Chlorophyll b; Total_ab: Total chlorophyll; Ratio_ab: Chlorophyll a/b; FBP: The flowering and boll-setting period; BOP: the boll opening period; AY: Anyang; HG: Huanggang; 16: 2016; 17: 2017. Asterisks indicate significance levels (***P < 0.001, **P < 0.01, and *P < 0.05).

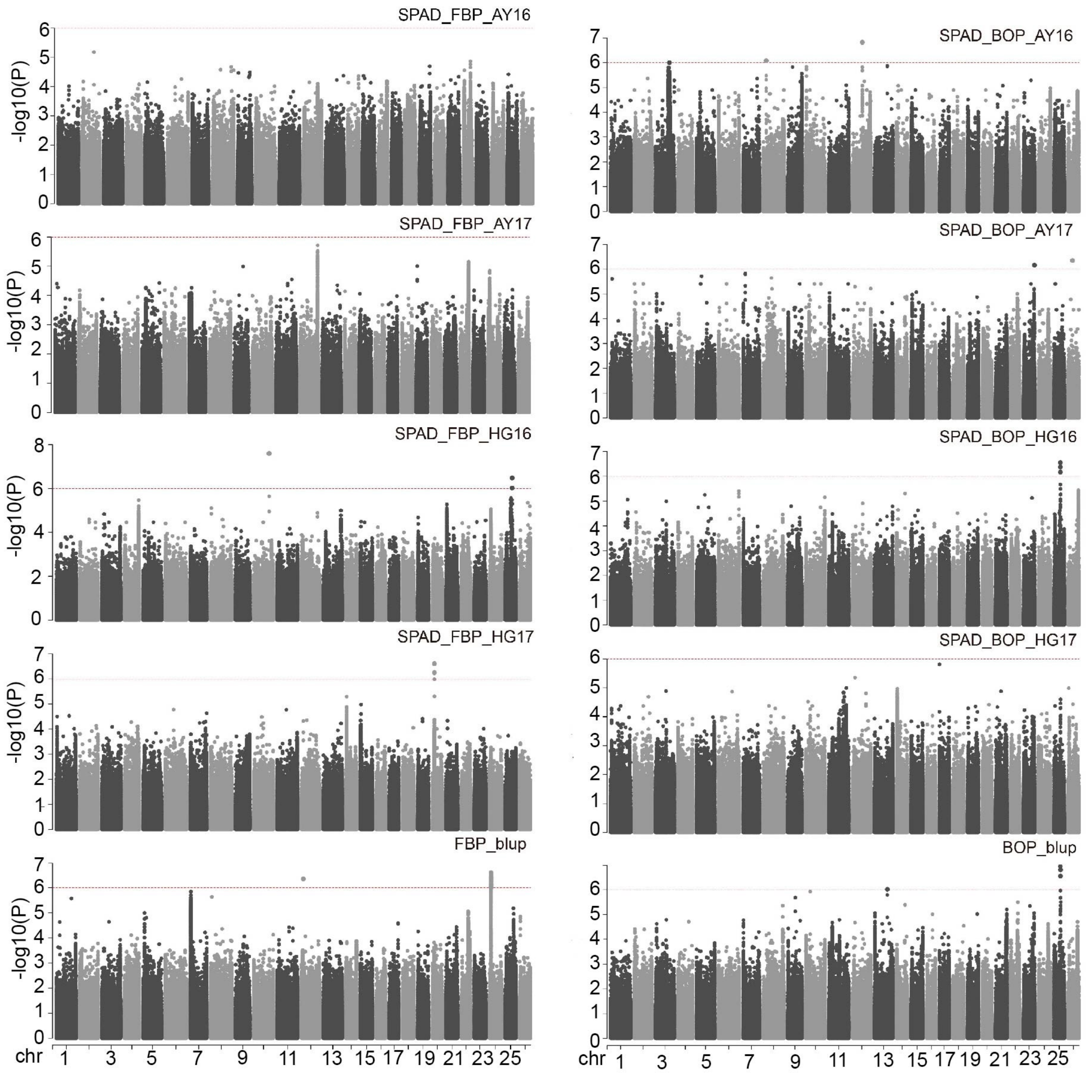

Figure S5. Manhattan plots of SAPD in each single environment and BLUP values. FBP: The flowering and boll-setting period; BOP: the boll opening period; blup: Best linear unbiased predictions; AY: Anyang; HG: Huanggang; 16: 2016; 17: 2017.

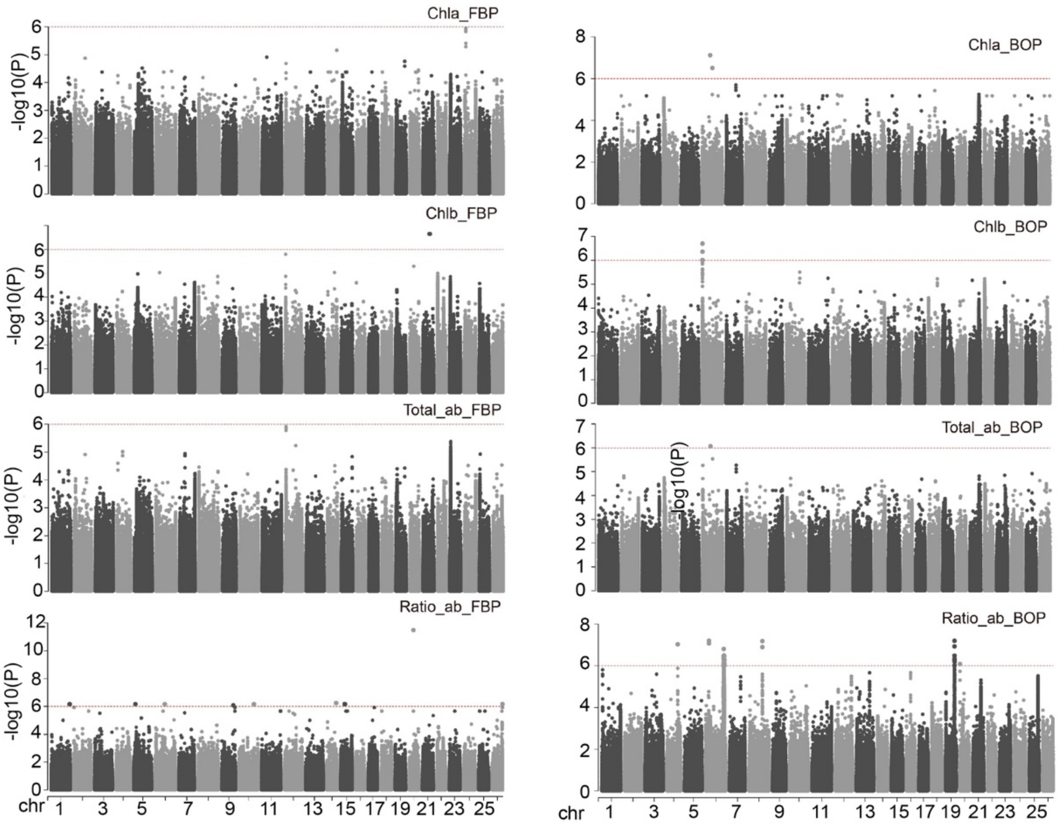

Figure S6. Manhattan plots of absolute chlorophyll concentrations at Anyang in 2017. D: the diurnal variation; Chla: Chlorophyll a; Chlb: Chlorophyll b; Total_ab: Total chlorophyll; Ratio_ab: Chlorophyll a/b; FBP: The flowering and boll-setting period; BOP: the boll opening period;

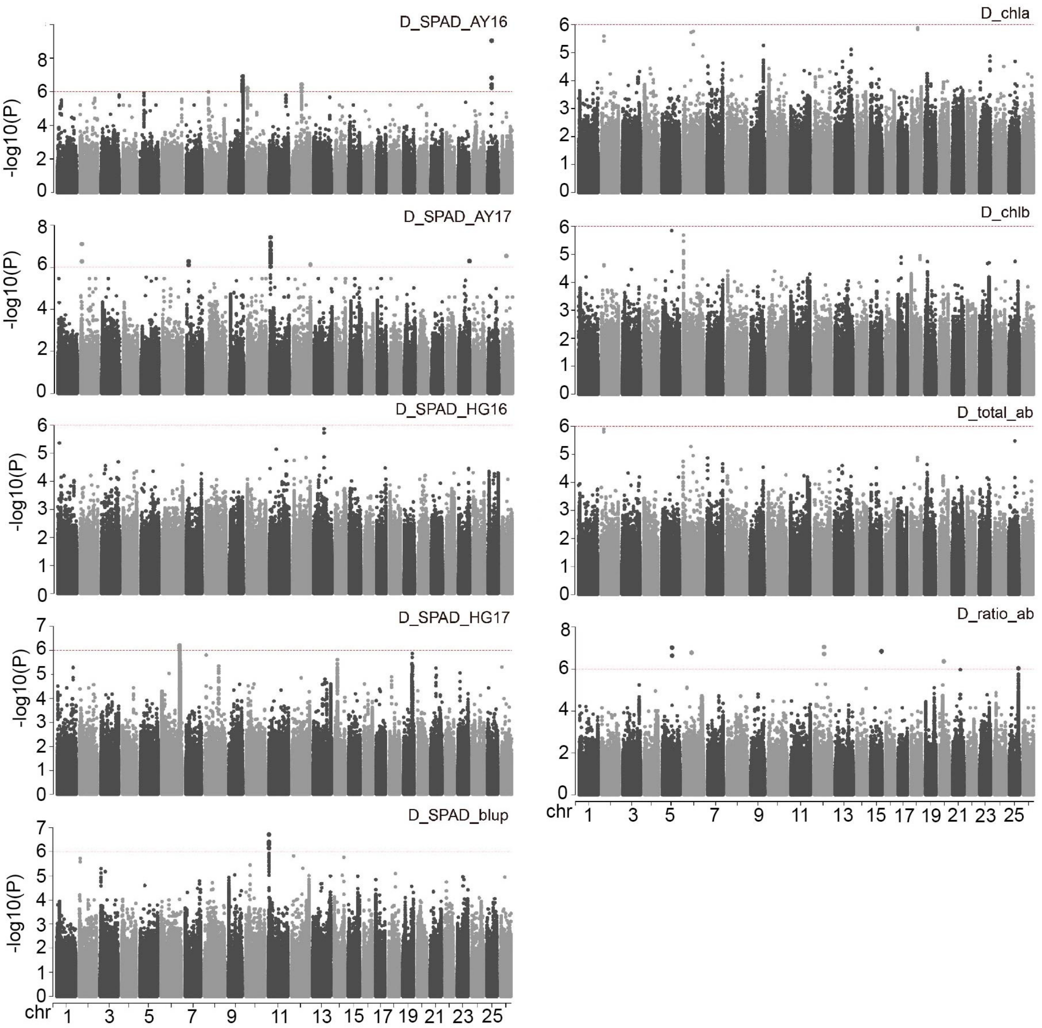

Figure S7. Manhattan plots of the diurnal variation of chlorophyll content. D: the diurnal variation; Chla: Chlorophyll a; Chlb: Chlorophyll b; Total_ab: Total chlorophyll; Ratio_ab: Chlorophyll a/b; blup: Best linear unbiased predictions; FBP: The flowering and boll-setting period; BOP: the boll opening period; AY: Anyang; HG: Huanggang; 16: 2016; 17: 2017.

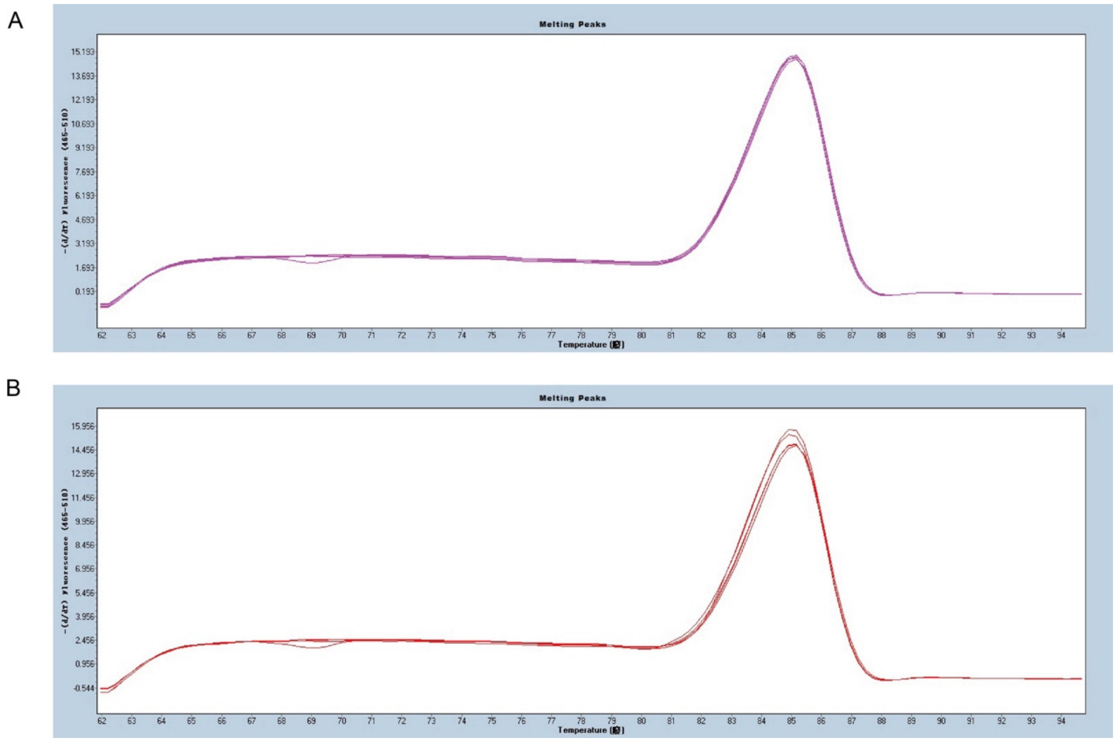

Figure S8. Melting curve analysis from qRT-PCR of *GhMKK9* gene. (A) and (B) represented the melting curves for cotton plants and transgenic *Arabidopsis* plants (OE7 and OE14), respectively. The single peak indicated the specific amplification.
